# Functional and structural MRI based obsessive-compulsive disorder diagnosis using machine learning methods

**DOI:** 10.1186/s12888-023-05299-2

**Published:** 2023-10-30

**Authors:** Fang-Fang Huang, Xiang-Yun Yang, Jia Luo, Xiao-Jie Yang, Fan-Qiang Meng, Peng-Chong Wang, Zhan-Jiang Li

**Affiliations:** 1 Department of Clinical Psychology, The National Clinical Research Center for Mental Disorders & Beijing Key Laboratory of Mental Disorders, Beijing Anding Hospital, Capital Medical University, Beijing, China; 2 Department of Preventive Medicine, College of Basic Medicine and Forensic Medicine, Henan University of Science and Technology, Henan, China

**Keywords:** Obsessive-compulsive disorder, Functional magnetic resonance imaging, Structural magnetic resonance imaging, Diagnosis model, Support vector machine

## Abstract

**Background:**

The success of neuroimaging in revealing neural correlates of obsessive-compulsive disorder (OCD) has raised hopes of using magnetic resonance imaging (MRI) indices to discriminate patients with OCD and the healthy. The aim of this study was to explore MRI based OCD diagnosis using machine learning methods.

**Methods:**

Fifty patients with OCD and fifty healthy subjects were allocated into training and testing set by eight to two. Functional MRI (fMRI) indices, including amplitude of low-frequency fluctuation (ALFF), fractional ALFF (fALFF), regional homogeneity (ReHo), degree of centrality (DC), and structural MRI (sMRI) indices, including volume of gray matter, cortical thickness and sulcal depth, were extracted in each brain region as features. The features were reduced using least absolute shrinkage and selection operator regression on training set. Diagnosis models based on single MRI index / combined MRI indices were established on training set using support vector machine (SVM), logistic regression and random forest, and validated on testing set.

**Results:**

SVM model based on combined fMRI indices, including ALFF, fALFF, ReHo and DC, achieved the optimal performance, with a cross-validation accuracy of 94%; on testing set, the area under the receiver operating characteristic curve was 0.90 and the validation accuracy was 85%. The selected features were located both within and outside the cortico-striato-thalamo-cortical (CSTC) circuit of OCD. Models based on single MRI index / combined fMRI and sMRI indices underperformed on the classification, with a largest validation accuracy of 75% from SVM model of ALFF on testing set.

**Conclusion:**

SVM model of combined fMRI indices has the greatest potential to discriminate patients with OCD and the healthy, suggesting a complementary effect of fMRI indices on the classification; the features were located within and outside the CSTC circuit, indicating an importance of including various brain regions in the model.

**Supplementary Information:**

The online version contains supplementary material available at 10.1186/s12888-023-05299-2.

## Background

Obsessive-compulsive disorder (OCD) is a common psychiatric condition with a lifetime prevalence between 1 and 3% in the general population [1, 2]. It is characterized by obsessions and/or compulsions with a continuous course if untreated [3]. OCD is among the most disabling psychiatric disorders [4], which leads to an enormous impairment in quality of life [5, 6] and constitutes a major health-economic burden on society [2, 7]. In recent years, magnetic resonance imaging (MRI) studies have provided numerous evidence of functional and structural abnormalities in various brain regions in OCD, mainly within the cortico-striato-thalamo-cortical (CSTC) circuit [8–12]. The success of neuroimaging in revealing the neural correlates of OCD has raised hopes of using MRI indices to discriminate OCD patients and the healthy.

In order to identify the neuroimaging biomarkers and implement the diagnosis classification, machine learning methods have been introduced. Machine learning methods have the advantage of being able to detect subtle and spatially distributed effects of neuroimaging data [13] and allow inference at the individual level rather than the group [14]. For example, support vector machine (SVM), one of the most widely used machine learning models, has been applied to build the diagnosis model of psychiatric disorders, such as schizophrenia [15, 16], depression [17, 18] and autism [19, 20]. Therefore, in this study, we applied MRI data and machine learning methods, including SVM and other two widely used classifiers, that is logistic regression (LR) and random forest (RF), to discriminate OCD patients and healthy subjects.

To be specific, amplitude of low-frequency fluctuation (ALFF), fractional ALFF (fALFF), regional homogeneity (ReHo) and degree of centrality (DC) were extracted from resting-state functional MRI (rs-fMRI) as the functional neuroimaging markers; volume of gray matter (VGM), cortical thickness and sulcal depth were extracted from T1-weighted images as the structural neuroimaging markers. These MRI indices were used because previous studies have successfully revealed altered ALFF [21–23], fALFF [24–27], ReHo [28–31], DC [32, 33], VGM [34–36] and cortical thickness [37–39] in various brain regions, including the traditional CSTC circuit and newly reported regions, such as the occipital, parietal, and temporal lobes and the cerebellum, in patients with OCD. Sulcal depth may provide valuable information for classification since previous studies indicate the association of altered sulcus morphology and psychotic disease [40–42]. In addition, these functional MRI (fMRI) and structural MRI (sMRI) indices can be easily calculated and explained, with no need for a priori selection of brain region like functional connectivity, as such has an advantage of clinical application.

Furthermore, it is unknown which index can achieve the optimal performance among these fMRI and sMRI indices, and whether a combination of multilevel MRI indices can improve the classification performance. At present, only a small number of studies applied fMRI or sMRI data to discriminate patients with OCD and healthy subjects, which mainly focused on separate index [43–48]. For example, one study found training of an SVM classifier to distinguish OCD patients from healthy subjects achieved excellent performance when using ALFF maps and good performance when using ReHo maps, with cross-validation accuracy of 95.37% and 86.11%, respectively [43]. Another study applied VGM to discriminate OCD patients and healthy subjects, achieving a cross-validation accuracy of 75.76% [44].

It is also important to explain the contribution of the MRI markers when constructing the OCD diagnosis models. However, due to the “black box” problem of machine learning models, such as SVM, previous studies seldom explored the contribution of the MRI markers used in the classification models. The Shapley value is a fair profit allocation among many stakeholders depending on their contribution and was derived from the name of the economist who introduced it. By using the idea of the Shapley value, approaches were proposed to interpret the predictions from any “black box” model [49, 50]. The key component of general explanations is the contributions (equivalent to the Shapley value) of individual input features. A prediction is explained by assigning to each feature a number which denote its influence. For each feature, such contributions can be aggregated to plot the feature’s average contribution against the feature’s value. This provides an overview of the model and explanation of the predictions.

Therefore, the aim of this study was to build and validate classification models based on fMRI and sMRI indices to discriminate patients with OCD and healthy subjects. Specifically, first, to investigate which MRI index achieves the optimal performance; second, to investigate whether a combination of multilevel MRI indices improves the performance of the classification; and third, to investigate the contribution of the MRI markers on classification.

## Methods

### Subjects

Fifty patients with OCD and 50 healthy control subjects (HCS) were included in this study. The patients and healthy subjects were recruited from Beijing Anding Hospital and local community, respectively. All the participants were diagnosed and classified using Structured Clinical Interview for Diagnostic and Statistical Manual of Mental Disorders, Fourth Edition (DSM-IV) Axis I Disorders (SCID), Patient Edition and Nonpatient Edition. The inclusion criteria for patients with OCD were as follows: (1) right-hand, (2) 18–60 years old, (3) Yale-Brown Obsessive-Compulsive Scale (Y-BOCS) score ≥ 16, (4) 17-item Hamilton Depression Rating Scale (HAMD-17) score < 18. The exclusion criteria for patients with OCD were as follows: (1) having taken or were taking psychiatric or psychological treatment, (2) having other mental disorders, neurological illnesses or major physical diseases. The inclusion criteria for healthy subjects were as follows: (1) right-hand, (2) 18–60 years old, (3) no history of any mental disorders, neurological illnesses, or major physical diseases.

### MRI acquisition

All the subjects were scanned on a Siemens Trio 3-Tesla scanner with a 32-channel head coil. T1-weighted images were obtained using a sagittal 3D magnetization prepared rapid gradient echo (MP-RAGE) sequence: slice number = 144, time repetition (TR) = 2530 ms, time echo (TE) = 3.39 ms, flip angle (FA) = 7°, slice thickness = 1.33 mm, field of view (FOV) = 256 × 256 mm^2^, in-plane resolution = 256 × 256, time to inversion (TI) = 1100 ms, voxel size = 1 × 1 × 1.33 mm^3^. The resting-state functional images were obtained using an echo-planar imaging (EPI) sequence (8 min): slice number = 33, thickness/gap = 3.5/0.6 mm, TR = 2000 ms, TE = 30 ms, FOV = 200 × 200 mm^2^, in-plane resolution = 64 × 64, FA = 90°, and 200 volumes.

### Preprocessing and feature extraction for rs-fMRI data

DPABI [51] was used for rs-fMRI data preprocessing with the following steps: removing the first 10 time points; slice timing; head motion correction; co-registered T1 images to functional images; segmentation with DARTEL; nuisance covariates regression (Friston 24, white matter and cerebrospinal fluid); normalization by DARTEL; and detrend. All the subjects met the head motion criteria of less than 2° of maximal rotation and 2 mm of maximal translation. ALFF [52], fALFF [53], ReHo [54] and DC [55] were extracted using DPABI. After preprocessing and smoothing, mean ALFF and fALFF maps were generated. ALFF detects the neural fluctuations within 0.01–0.08 Hz, reflecting intensity of regional spontaneous brain activity. fALFF is a ratio of amplitude within 0.01–0.08 Hz to the total amplitude within the full frequency band, indicating the relative contribution of spontaneous brain activity. After preprocessing and filter, mean ReHo and mean weighted DC with correlation coefficient > 0.25 were calculated and smoothed with a Gaussian kernel of 4 mm. ReHo reflects regional brain activity by calculating the Kendall coefficient of concordance between a particular voxel and its nearby neighbors (26 voxels). Weighted DC is defined as the sum of weights from edges connecting to a node and represents the node strength. We eventually obtained 116 ALFF, 116 fALFF, 116 ReHo and 116 DC features by averaging all the voxels in each region of interest based on Anatomical Automatic Labeling (AAL) atlas. Thus, a total of 464 fMRI features were extracted.

### Preprocessing and feature extraction for sMRI data

CAT12 toolbox for SPM12 was used for sMRI data preprocessing with the following steps: segmentation with a prior tissue probability map; calculating total intracranial volume (TIV) in native-space; registering native-space segmentations to a standard Montreal Neurological Institute (MNI) template; modulation to mitigate volume changes caused by spatial normalization. Finally, VGM, cortical thickness, and sulcal depth were extracted using CAT12. A total of 412 sMRI features were extracted based on the atlas. Among of them, 116 VGM features were obtained based on AAL atlas, 148 cortical thickness and 148 sulcal depth features were obtained based on Destrieux aparc.a2009s atlas [56].

### Feature selection

The whole dataset was divided into training set and testing set according to the number sequence of the subjects. The first 80% of the participants (including 40 patients with OCD and 40 HCS) were selected as the training set and the last 20% of the participants (including 10 patients with OCD and 10 HCS) were selected as the testing set. We used the same training set to select features and train classifiers. The testing set was used to validate the performance of the classification models.

Least absolute shrinkage and selection operator (LASSO) logistic regression was conducted to select features. LASSO is a popular method for regression that uses an L1 penalty to achieve a sparse solution and shrinks the coefficient estimates toward zero, with the degree of shrinkage dependent on an additional penalty parameter, lambda (λ) [57, 58]. The L1 penalty is the sum of the absolute coefficients (${w}_{j}$): ${\left|w\right|}_{1}={\sum }_{j=1}^{p}\left|{w}_{j}\right|$. LASSO uses this L1 penalty by adding λ to control the penalization: $\widehat{w}={arg}\underset{w}{{min}}{\sum }_{i=1}^{n}{({y}_{i}-{\sum }_{j}{x}_{ij}{w}_{j})}^{2}+\lambda {\sum }_{j=1}^{p}\left|{w}_{j}\right|.$ Firstly, LASSO regression was conducted to select inputting variables for single MRI index models. Then, the selected features of each index were reduced again by LASSO for the combined MRI indices models.

We utilized software R (version 4.3.1), package “glmnet” (version 4.1-7) to fit the LASSO regression. The optimal λ was determined through leave-one-out cross-validation (LOOCV), which means leaving one subject out as the testing data, and the others as the training data. The binomial deviance was computed as the measure to be minimized when cross-validating the selected model. Model with a lower deviance fits better. The cross-validation produces two optional λ values, λ_min_ and λ_1se_. λ_min_ minimizes the average binomial deviance of LOOCV and λ_1se_ represents largest λ that is still within one standard error of the minimum binomial deviance. λ_min_ was considered as the optimal λ in this study because it results in weaker penalty than λ_1se_, thus allowing us to include enough features. After obtaining the optimal λ, the LASSO regression was fitted again for all the subjects using the selected λ and features with nonzero coefficient were retained.

### Classification and validation

Radial basis function (RBF) kernel SVM, LR and RF classifiers were used to build the classification models on training set (including 40 patients with OCD and 40 HCS). Classification models based on single MRI index and combined MRI indices were established. We utilized software R (version 4.3.1), package “caret” (version 6.0–94) to fit the RBF kernel SVM, LR and RF models.

There are two tuning parameters for RBF kernel SVM in “caret”, sigma and cost (C). We used on the function “sigest” from the package “kernlab” (version 0.9–32) to estimate the range of values for the sigma parameter which would return good results when used with SVM models [59]. The estimation is based upon the 0.1 and 0.9 quantile of $||x - x'|{|^2}$. Basically, any value in between those two bounds will produce good results. Then sigma and C were tuned by the grid search through LOOCV. The grid search contained 54 combinations with six sigma values ranging from minimum sigma to maximum sigma generated by function “sigest”, and nine C values (0.25, 0.5, 1, 2, 4, 8, 16, 32, 64). The hyperparameter (mtry) of RF was tuned by the grid search of 1 to *k* (*k* = number of features) through LOOCV. Area under the receiver operating characteristic (ROC) curve (AUC) was used to select the optimal model using the largest value of AUC.

Average accuracy, sensitivity and specificity of LOOCV were calculated to show the performance of fitted models on training set. Then, we validated the performance of the classification models on testing set (including 10 patients with OCD and 10 HCS). Accuracy, sensitivity and specificity were calculated and ROC curves with AUC values were drawn for the classification models on testing set.

### The contribution of the features

We investigated the contribution of features for the classification model that obtained optimal performance. We used software R (version 4.3.1), package “fastshap” (version 0.1.0) to compute fast approximate Shapley values of features for each individual, and visualized the mean absolute Shapley value of all the subjects to show the contribution of the feature. A higher mean absolute Shapley value indicates a larger contribution to the classification.

### Other statistical analyses

Independent sample *t* test was used to compare the characteristics of subjects between OCD and HCS group. A two-tailed *P* value < 0.05 was considered significant. software R (version 4.3.1) was used to conduct these statistical analyses. BrainNet viewer [60] was used to visualize the brain regions.

## Results

### Basic information of participants

Basic information of subjects is shown in Table 1. A total of 40 patients with OCD and 40 HCS were included in the training set; a total of 10 patients with OCD and 10 HCS were included in the testing set. There were no statistically significant differences in age, sex, head motion or TIV between patients with OCD and HCS (*P* > 0.05) in both training and testing set. The mean Y-BOCS and HAMD score of patients with OCD in training set was 23.85 and 6.60, respectively; the mean Y-BOCS and HAMD score of patients with OCD in testing set was 23.50 and 6.80, respectively.

**Table 1:** Basic information of participants

Dataset	Variables	OCD			HCS			
		Mean	SD		Mean	SD	*t* /${\chi }^{2}$	*P*
Training	Age (years)	28.80	6.91		28.48	6.01	2.224	0.823
	Head motion ^a^	0.08	0.03		0.08	0.04	–0.839	0.404
	TIV (cm^3^)	1489	139		1461	107	0.991	0.325
	Sex (F/M) ^b^	13/27			14/26		0.056	0.813
	HAMD	6.60	4.19		/	/	/	/
	Y-BOCS	23.85	5.34		/	/	/	/
Testing	Age (years)	30.00	8.19		28.50	8.91	3.392	0.700
	Head motion ^a^	0.07	0.03		0.07	0.03	–0.567	0.578
	TIV (cm^3^)	1459	148		1462	89	–0.051	0.960
	Sex (F/M) ^b^	5/5			4/6		0.202	0.653
	HAMD	6.80	4.54		/	/	/	/
	Y-BOCS	23.50	7.46		/	/	/	/

Notes: F, Female; HAMD, Hamilton Depression Scale; HCS, Healthy Control Subjects; M, Male; OCD, Obsessive-Compulsive Disorder; SD, Standard Deviation; TIV, Total Intracranial Volume; Y-BOCS, Yale-Brown Obsessive-Compulsive Scale. ^a^, Head motion was evaluated by mean FD Jenkinson; b, The difference was compared by ${\chi }^{2}$ test

### Features selection by LASSO

**Fig. 1:**
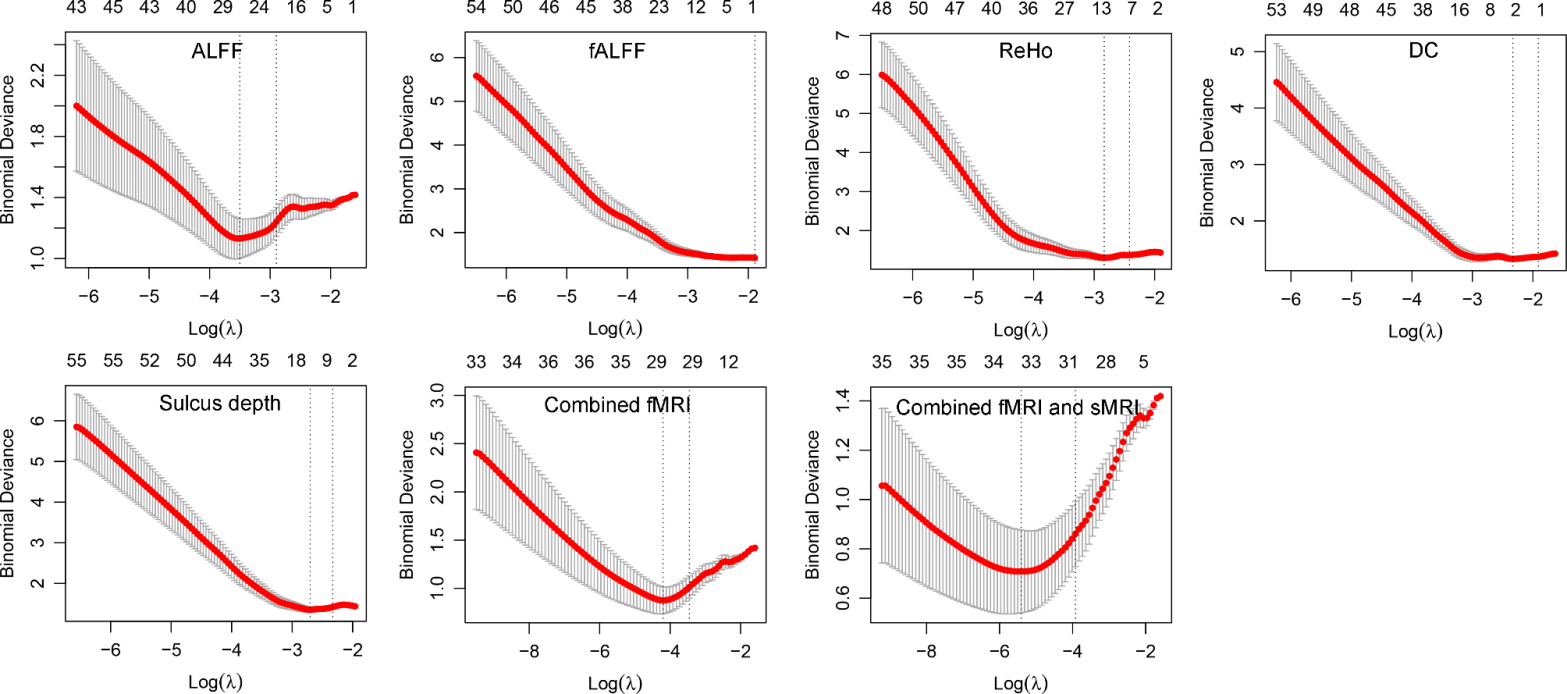
Optimal lambda selection of LASSO through cross-validation. Notes: ALFF, Amplitude of Low Frequency Fluctuation; DC, Degree of Centrality; fALFF, Fractional Amplitude of Low Frequency Fluctuation; fMRI, Functional Magnetic Resonance Imaging; ReHo, Regional Homogeneity; sMRI, Structural Magnetic Resonance Imaging. The longitudinal coordinate represents the binomial deviance and the upper and lower standard deviation. The left dashed line points to λ_min_ and the right dashed line points to λ_1se_

Figure 1 shows the optimal λ selection of LASSO through LOOCV. There were 27, 1, 12, 2, 12, 29, and 35 features selected for ALFF, fALFF, ReHo, DC, sulcal depth, combined fMRI indices (including 19 ALFF, 1 fALFF, 7 ReHo and 2 DC features) and combined fMRI and sMRI indices (including 16 ALFF, 1 fALFF, 8 ReHo, 1 DC, and 9 sulcal depth features). There were no VGM or cortical thickness features selected by LASSO. The optimal λ values used in LASSO for feature selection are shown in supplementary materials file 1. The selected features are shown in supplementary materials file 2.

### The performance of classification models based on single MRI index

**Fig. 2:**
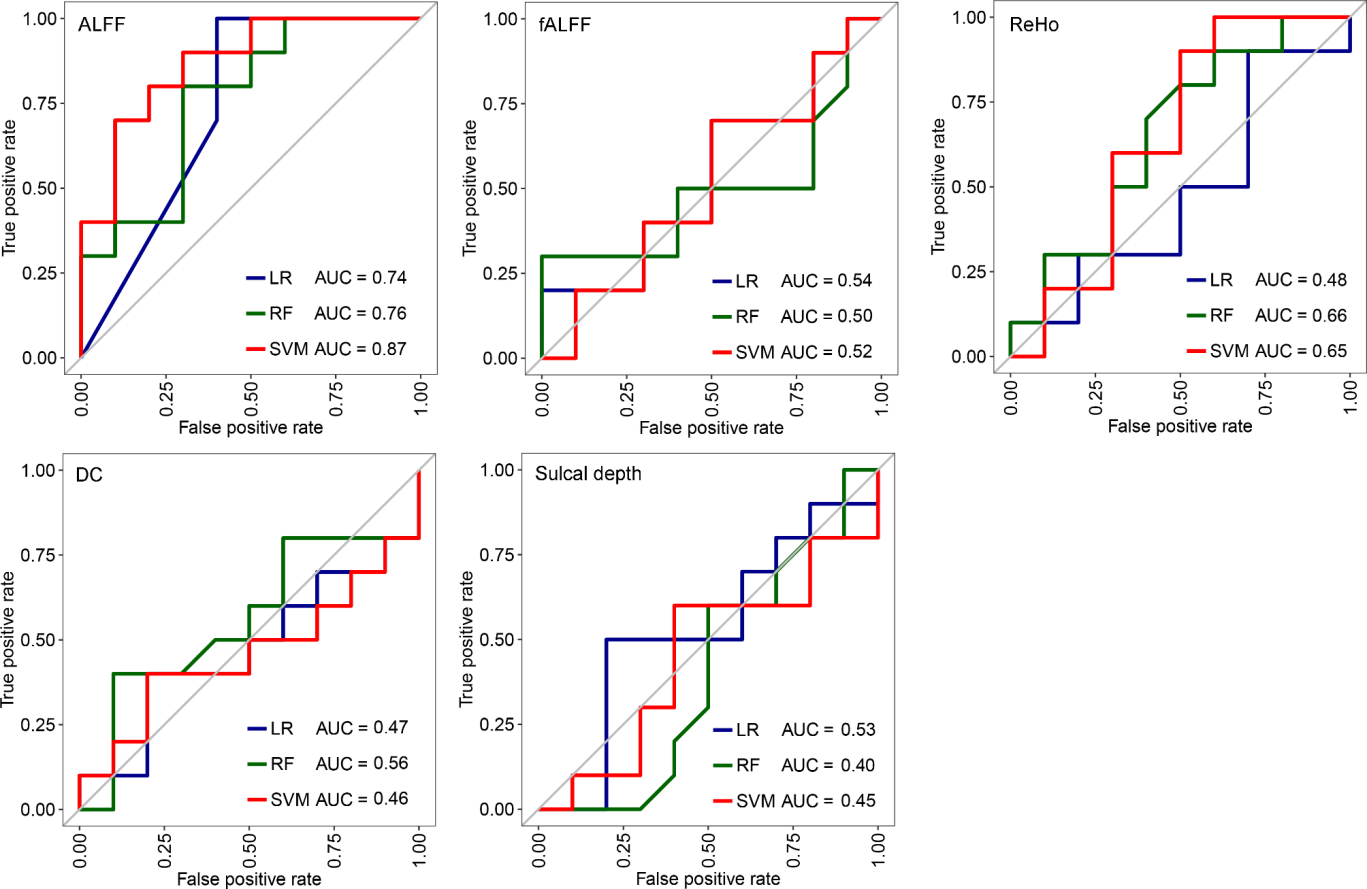
ROC curves of classification models based on single MRI index on testing set. Notes: ALFF, Amplitude of Low Frequency Fluctuation; AUC, Area Under the Curve; DC, Degree of Centrality; fALFF, Fractional Amplitude of Low Frequency Fluctuation; LR, Logistic Regression; ReHo, Regional Homogeneity; RF, Random Forest; SVM, Support Vector Machine

Table 2 exhibits the AUC, accuracy, sensitivity and specificity of classification models based on single MRI index. Figure 2 shows ROC curves of these classification models on testing set. The optimal hyperparameters used in SVM and RF models are shown in supplementary materials file 3. For single MRI index models, SVM model of ALFF achieved the best performance. On training set, the cross-validation AUC and accuracy was 0.98 and 93%, respectively; on testing set, the AUC was 0.87 and the accuracy was 75%. Although SVM model of ReHo and SVM model of sulcal depth achieved good performance by LOOCV on training set, with AUC values larger than 0.90, the performance on testing set was poor. Classification models of fALFF / DC yielded poor performance.

**Table 2:** Performance of classification models based on single MRI index

Model	Indices	Training set					Testing set			
		AUC	Acc	Sens	Spec		AUC	Acc	Sens	Spec
SVM	ALFF	0.98	0.93	0.85	1.00		0.87	0.75	0.60	0.90
	fALFF	0.63	0.59	0.53	0.65		0.52	0.50	0.50	0.50
	ReHo	0.92	0.83	0.83	0.83		0.65	0.55	0.50	0.60
	DC	0.70	0.62	0.60	0.63		0.46	0.45	0.50	0.40
	Sulcal depth	0.95	0.87	0.83	0.90		0.45	0.60	0.60	0.60
LR	ALFF	0.96	0.89	0.83	0.95		0.74	0.75	0.60	0.90
	fALFF	0.63	0.59	0.58	0.60		0.54	0.50	0.50	0.50
	ReHo	0.86	0.82	0.83	0.80		0.48	0.45	0.40	0.50
	DC	0.71	0.64	0.63	0.65		0.47	0.45	0.50	0.40
	Sulcal depth	0.82	0.75	0.75	0.75		0.53	0.55	0.60	0.50
RF	ALFF	0.86	0.78	0.78	0.78		0.79	0.65	0.50	0.80
	fALFF	0.58	0.62	0.60	0.63		0.50	0.40	0.30	0.50
	ReHo	0.77	0.69	0.68	0.70		0.66	0.65	0.60	0.70
	DC	0.60	0.57	0.58	0.55		0.56	0.55	0.70	0.40
	Sulcal depth	0.78	0.70	0.65	0.75		0.40	0.50	0.40	0.60

Notes: Acc, Accuracy; ALFF, Amplitude of Low Frequency Fluctuation; AUC, Area Under the Curve; DC, Degree of Centrality; fALFF, Fractional Amplitude of Low Frequency Fluctuation; LR, Logistic Regression; ReHo, Regional Homogeneity; RF, Random Forest; Sens, Sensitivity; Spec, Specificity; SVM, Support Vector Machine

### The performance of classification models based on combined MRI indices

**Fig. 3:**
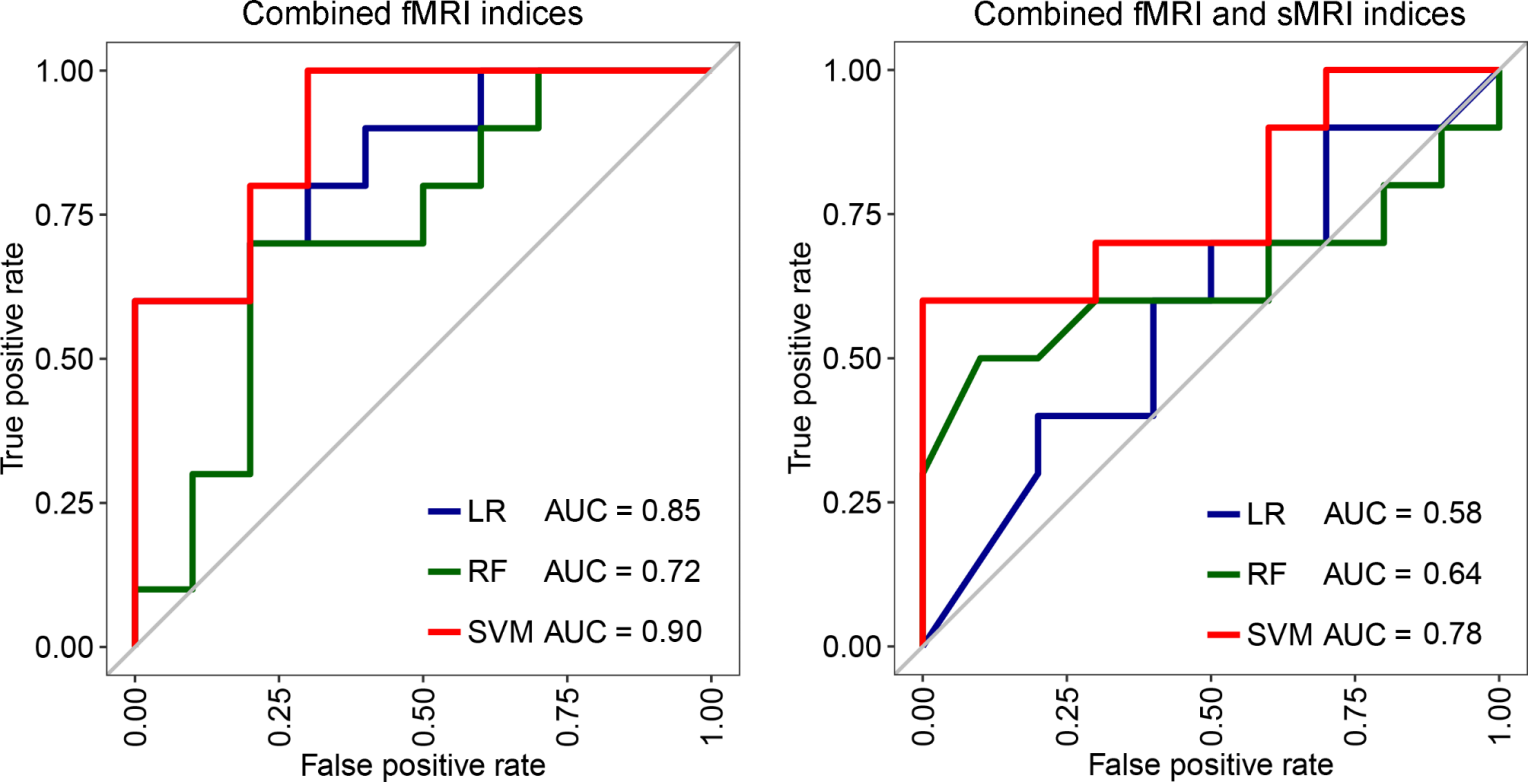
ROC curves of classification models based on combined MRI indices on testing set. Notes: AUC, Area Under the Curve; fMRI, Functional Magnetic Resonance Imaging; LR, Logistic Regression; RF, Random Forest; sMRI, Structural Magnetic Resonance Imaging; SVM, Support Vector Machine. fMRI indices included ALFF, fALFF, ReHo and DC; fMRI + sMRI indices included ALFF, fALFF, ReHo, DC and sulcal depth

Table 3 exhibits the AUC, accuracy, sensitivity and specificity of classification models based on combined MRI indices. Figure 3 shows ROC curves of these classification models on testing set. The optimal hyperparameters used in SVM and RF models are shown in supplementary materials file 3. SVM model of combined fMRI indices achieved excellent cross-validation performance on training set and good performance on testing set. On training set, the cross-validation AUC and accuracy was 0.99 and 94%, respectively; on testing set, the AUC was 0.90 and the accuracy was 85%. Classification models based on combined fMRI and sMRI indices underperformed on testing set. We did not construct combined sMRI indices models because only sulcal depth features were selected during the feature selection procedure.

**Table 3:** Performance of classification models based on combined MRI indices

Model	Indices	Training set					Testing set			
		AUC	Acc	Sens	Spec		AUC	Acc	Sens	Spec
SVM	fMRI	0.99	0.94	0.88	1.00		0.90	0.85	0.70	1.00
	fMRI + sMRI	1.00	0.97	0.93	1.00		0.78	0.60	0.50	0.70
LR	fMRI	0.88	0.81	0.83	0.78		0.85	0.70	0.60	0.80
	fMRI + sMRI	0.98	0.96	0.98	0.93		0.58	0.60	0.50	0.70
RF	fMRI	0.91	0.83	0.75	0.90		0.72	0.65	0.50	0.80
	fMRI + sMRI	0.96	0.89	0.85	0.93		0.64	0.50	0.40	0.60

Notes: Acc, Accuracy; AUC, Area Under the Curve; fMRI, Functional Magnetic Resonance Imaging; LR, Logistic Regression; RF, Random Forest; Sens, Sensitivity; sMRI, Structural Magnetic Resonance Imaging; Spec, Specificity; SVM, Support Vector Machine. fMRI indices included ALFF, fALFF, ReHo and DC; fMRI + sMRI indices included ALFF, fALFF, ReHo, DC and sulcal depth

### The contribution of features in SVM model based on combined fMRI indices

**Fig. 4:**
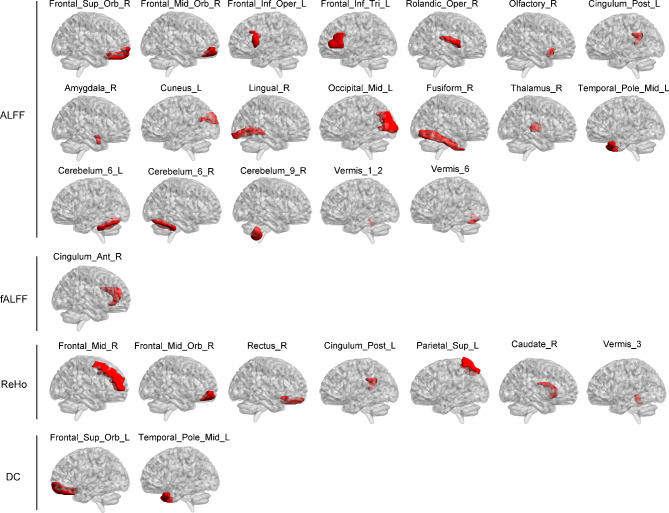
Features included in SVM model of combined fMRI indices. Notes: ALFF, Amplitude of Low Frequency Fluctuation; DC, Degree of Centrality; fALFF, Fractional Amplitude of Low Frequency Fluctuation; ReHo, Regional Homogeneity

**Fig. 5:**
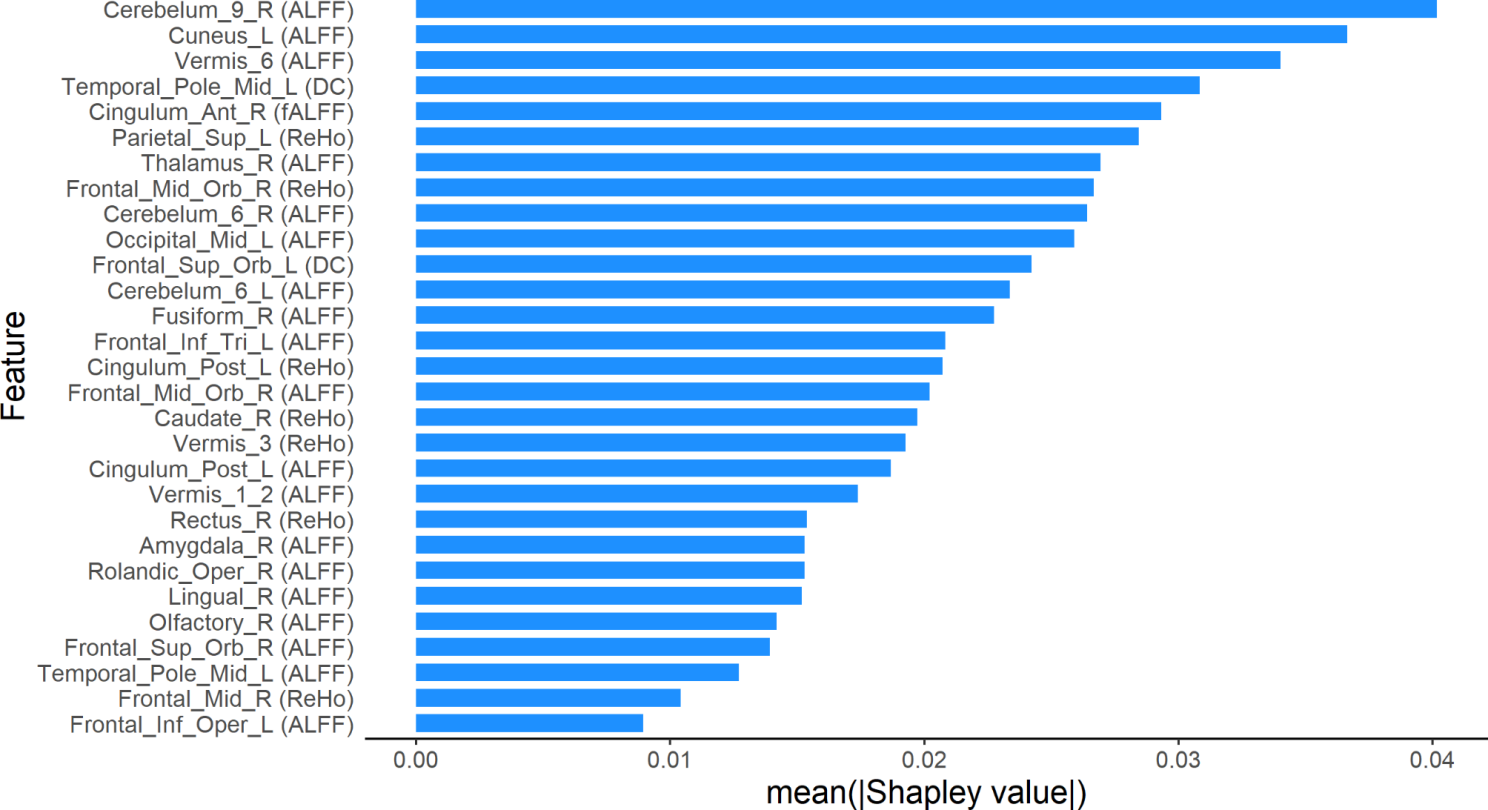
Contribution of features in SVM model of combined fMRI indices. Notes: ALFF, Amplitude of Low Frequency Fluctuation; DC, Degree of Centrality; fALFF, Fractional Amplitude of Low Frequency Fluctuation; ReHo, Regional Homogeneity

Figure 4 shows the location of the features included in SVM model of combined fMRI indices. Figure 5 shows the contribution of these features in the model. The top ten contribution came from ALFF of right Cerebelum_9_R, ALFF of left cuneus, ALFF of Vermis_6, DC of left temporal pole middle temporal gyrus, fALFF of right anterior cingulate and paracingulate gyri, ReHo of left superior parietal gyrus, ALFF of right thalamus, ReHo of right middle frontal gyrus orbital part, ALFF of right Cerebelum_6_R, and ALFF of left middle occipital gyrus.

The middle ten contribution came from DC of left superior frontal gyrus orbital part, ALFF of left Cerebelum_6_L, ALFF of right fusiform gyrus, ALFF of left inferior frontal gyrus triangular part, ReHo of left posterior cingulate gyrus, ALFF of right middle frontal gyrus orbital part, ReHo of right caudate nucleus, ReHo of Vermis_3; ALFF of left posterior cingulate gyrus, and ALFF of Vermis_1_2.

The bottom nine contribution came from ReHo of right gyrus rectus, ALFF of right amygdala, ALFF of right Rolandic operculum, ALFF of right lingual gyrus, ALFF of right olfactory cortex, ALFF of right superior frontal gyrus orbital part, ALFF of left temporal pole middle temporal gyrus, ReHo of right middle frontal gyrus, and ALFF of left inferior frontal gyrus opercular part.

## Discussion

To the best of our knowledge, this is the first study to comprehensively utilize multilevel MRI indices to distinguish OCD patients and healthy subjects. We applied different classifiers to build the classification models. SVM model based on combined fMRI indices, including ALFF, fALFF, ReHo and DC, exhibited optimal classification performance.

For single MRI index, ALFF achieved better performance than fALFF, ReHo, DC and sulcal depth. Bu et al., (2019) compared the classification performance of different rs-fMRI index and found that SVM model using voxel-wise ALFF map achieved the best performance on OCD diagnosis [43]. They argued that ALFF directly correlates with the intensity of spontaneous neural activity in the resting-state and is related to the rate of regional glucose metabolism, which could make ALFF more sensitive to detect dysfunctional neural activity than the other functional parameters [61].

Although previous studies have reported VGM [34–36] and cortical thickness [37–39] changes in patients with OCD compared with healthy controls, the VGM and cortical thickness features were not retained during the feature selection stage in our study. Some sulcal depth features were selected, however, the classification models of sulcal depth underperformed on testing set. Previous studies also showed limited accuracy when using structural neuroanatomy and machine learning methods to build diagnosis model of OCD [44, 47]. Considering the current results and previous findings, the value of using single sMRI index to distinguish OCD and the healthy needs to be further verified.

Although ALFF exhibited stronger classification power than other MRI indices, the performance of ALFF models on testing set was less than satisfactory. The results indicate that single MRI index cloud not provide enough information to discriminate patients with OCD and the healthy. ALFF, fALFF, ReHo, DC and sulcal depth reflect the neuroimaging changes of OCD from different perspective. ALFF reflects intensity of regional spontaneous brain activity [52]; fALFF reflects the relative contribution of the oscillations [53]; ReHo represents local coherence of spontaneous brain activity [54]; weighted DC shows functional connectivity strength of a certain brain region to the whole brain [55]; sulcal depth has been widely used to study the morphological characteristic of the cerebral folding [62, 63]. It seems to be a reasonable hypothesis that the combination of multilevel MRI indices can improve the classification performance.

As expected, a combination of multilevel fMRI indices improved the classification performance compared with single MRI index, indicating a complementary effect of ALFF, fALFF, ReHo and DC on the classification of OCD and HCS. Unfortunately, classification models based on combined fMRI and sMRI indices underperformed on testing set. The results suggest that sMRI index (that is sulcal depth in this study) may have an interference effect on fMRI indices for the classification of OCD and HCS.

For different classifiers, SVM exhibited superior performance than LR and RF in this study. SVM aims to classify data points by maximizing the margin between classes in a high dimensional space [14]. Evidence of comparison among machine learning approaches showed that SVM helps weigh down the effect of noisy features that are highly correlated with each other when there are a large number of features [64] and outperforms other machine learning classifiers on MRI-based brain tumor [65] and autism classification [20].

In SVM model of combined fMRI indices, the features were located both within the traditional CSTC circuit of OCD (such as anterior cingulate cortex, thalamus, orbitofrontal cortex, prefrontal cortex, caudate nucleus), and outside the CSTC circuit (distributing among parietal, temporal, occipital cortex and cerebellum). It is consistent with researches which investigated the neuroimaging alterations in patients with OCD. The dysfunction of CSTC circuit has been classically considered to underpin the clinical manifestations of OCD [9, 10, 12]. However, in recent years, evidence has been accumulating pointing to other regions outside the CSTC circuit, such as parietal, temporal, occipital cortex and cerebellum [27, 66, 67]. Furthermore, in top / middle / bottom contribution group, the features were also located both within and outside the CSTC circuit of OCD. The results suggest that future researches need to consider the role of indicators in various brain regions, as well as to further explore the neurophysiological mechanisms of OCD.

### Limitation and future direction

Despite the novel findings, it is important to note limitations within this study. First, this study was designed and analyzed on relatively small, single center data. Second, we included patients with diagnostic OCD without any comorbidities or prior treatment history, which may decrease the generalizability of our findings. In the real world, the diagnosis of OCD is much more complicated. Some individuals may experience obsessive-compulsive symptom, however, not meet the diagnostic criteria. Therefore, the explanation of the models in this study should be limited to diagnostic OCD without comorbidities and treatment history. Therefore, including a larger, multicenter sample with different clinical characteristics to validate the generalizability of the model is needed.

For the future direction, developing a computer-aid neuroimaging automated diagnosis system to implement outputs of diagnosis from inputs of MRI images is worth exploring. The procedure should include MRI scanning, raw images preprocessing, feature extracting, representation inputting and diagnosis outputting. Some challenges and restrictions should be considered. First, the patients have to take MRI scanning using the same sequence with the same parameter setting; second, current preprocessing and features extracting are based on manual manipulation in the software and usually time-consuming; third, it is better to develop an automated diagnosis software which can connect the different steps to simplify the computer-aid diagnosis process.

## Conclusion

In summary, SVM model of combined fMRI indices, including ALFF, fALFF, ReHo and DC, exhibited good performance on discriminating OCD patients and the healthy. The selected brain regions in SVM model of combined fMRI indices were located both within and outside the traditional CSTC circuit of OCD.

### Electronic supplementary material

Below is the link to the electronic supplementary material.

Supplementary Material 1

Supplementary Material 2

Supplementary Material 3

## Data Availability

The datasets generated and / or analyzed during the current study are not publicly available due to exposition of subjects’ private information, but are available from the corresponding author on reasonable request.

## List of abbreviations

AAL atlas: Anatomical Automatic Labeling atlas
ALFF: Amplitude of low-frequency fluctuation
AUC: Area under the curve
CSTC circuit: Cortico-striato-thalamo-cortical circuit
DC: Degree of centrality
fALFF: Fractional amplitude of low-frequency fluctuation
fMRI: Functional magnetic resonance imaging
HAMD: Hamilton depression rating scale
HCS: Healthy control subjects
LASSO: Least absolute shrinkage and selection operator
LR: Logistic regression
LOOCV: Leave-one-out cross-validation
MNI: Montreal Neurological Institute
MRI: Magnetic resonance imaging
OCD: Obsessive-compulsive disorder
RBF: Radial basis function
ReHo: Regional homogeneity
RF: Random forest
ROC curve: Receiver operating characteristic curve
rs-fMRI: Resting-state functional magnetic resonance imaging
SD: Standard deviation
sMRI: Structural magnetic resonance imaging
SVM: Support vector machine
TIV: Total intracranial volume
Y-BOCS: Yale-brown obsessive-compulsive scale
VGM: Volume of gray matter
